# Dynamic Transcriptome Profiling of Mungbean Genotypes Unveil the Genes Respond to the Infection of Mungbean Yellow Mosaic Virus

**DOI:** 10.3390/pathogens11020190

**Published:** 2022-01-30

**Authors:** Manickam Sudha, Adhimoolam Karthikeyan, Balasubramaniam Madhumitha, Rajagopalan Veera Ranjani, Mayalagu Kanimoli Mathivathana, Manickam Dhasarathan, Jayakodi Murukarthick, Madiha Natchi Samu Shihabdeen, Karuppiah Eraivan Arutkani Aiyanathan, Muthaiyan Pandiyan, Natesan Senthil, Muthurajan Raveendran

**Affiliations:** 1 Department of Plant Biotechnology, Centre for Plant Molecular Biology and Biotechnology, Tamil Nadu Agricultural University, Coimbatore 641003, Tamil Nadu, India; rajaranji@gmail.com (R.V.R.); madihasamu16@gmail.com (M.N.S.S.); raveendrantnau@gmail.com (M.R.); 2 Department of Biotechnology, Centre of Innovation, Agricultural College and Research Institute, Tamil Nadu Agricultural University, Madurai 625104, Tamil Nadu, India; karthik2373@gmail.com; 3 Department of Plant Pathology, Agricultural College and Research Institute, Tamil Nadu Agricultural University, Madurai 625104, Tamil Nadu, India; madhu2sucess@gmail.com; 4 Department of Plant Breeding and Genetics, Agricultural College and Research Institute, Tamil Nadu Agricultural University, Madurai 625104, Tamil Nadu, India; kani_vathana@yahoo.co.in; 5 Agroclimate Research Centre, Directorate of Crop Management, Tamil Nadu Agricultural University, Coimbatore 641003, Tamil Nadu, India; plantdr.dhasarathan@gmail.com; 6 Gene Bank, Leibniz Institute of Plant Genetics and Crop Plant Research (IPK) Stadt See land, 06466 Seeland, OT Gatersleben, Germany; jayakodi@ipk-gatersleben.de; 7 Agricultural College and Research Institute, Tamil Nadu Agricultural University, Killikulam 628252, Tamil Nadu, India; eraivan@rediffmail.com; 8 Regional Research Station, Tamil Nadu Agricultural University, Virudhachalam 606001, Tamil Nadu, India; mpandiyan8@yahoo.co.in; 9 Department of Plant Molecular Biology and Bioinformatics, Centre for Plant Molecular Biology and Biotechnology, Tamil Nadu Agricultural University, Coimbatore 641003, Tamil Nadu, India; senthil_natesan@tnau.ac.in

**Keywords:** mungbean, RNA seq, whitefly, yellow mosaic disease

## Abstract

Yellow mosaic disease (YMD), incited by mungbean yellow mosaic virus (MYMV), is a primary viral disease that reduces mungbean production in South Asia, especially in India. There is no detailed knowledge regarding the genes and molecular mechanisms conferring resistance of mungbean to MYMV. Therefore, disclosing the genetic and molecular bases related to MYMV resistance helps to develop the mungbean genotypes with MYMV resistance. In this study, transcriptomes of mungbean genotypes, VGGRU-1 (resistant) and VRM (Gg) 1 (susceptible) infected with MYMV were compared to those of uninfected controls. The number of differentially expressed genes (DEGs) in the resistant and susceptible genotypes was 896 and 506, respectively. Among them, 275 DEGs were common between the resistant and susceptible genotypes. Functional annotation of DEGs revealed that the DEGs belonged to the following categories defense and pathogenesis, receptor-like kinases; serine/threonine protein kinases, hormone signaling, transcription factors, and chaperons, and secondary metabolites. Further, we have confirmed the expression pattern of several DEGs by quantitative real-time PCR (qRT-PCR) analysis. Collectively, the information obtained in this study unveils the new insights into characterizing the MYMV resistance and paved the way for breeding MYMV resistant mungbean in the future.

## 1. Introduction 

Yellow mosaic disease (YMD) is a major virus disease, and its incidence has become severe in the past three decades throughout most mungbean (*Vigna radiata*) producing regions in South Asia and particularly in India. Three different begomoviruses, i.e., mungbean yellow mosaic virus (MYMV), mungbean yellow mosaic India virus (MYMIV), and horse gram yellow mosaic virus (HgYMV), have been found to cause YMD in various mungbean producing regions of Asia [1]. MYMV and MYMIV cause the YMD in India. It was reported that MYMV is primarily in India’s southern region, while MYMIV is in the northern, central, and eastern regions of India [2,3]. The viruses are spread by whitefly in a persistent and circulative manner but cannot be transmitted mechanically through sap or seed. The effectiveness of whitefly transmission and behavior varies with that of the genotype and growth stage and virus strains [4]. The most common virus symptoms include yellowing or chlorosis of the leaves preceded by necrosis, fewer flowers and pods, and pods containing immature and abnormal seeds and stunting plants. Mungbean plants infected within 3 weeks of sowing may reduce yield up to 85%. So far, many investigations on the mungbean-MYMV have concentrated on the occurrence, characterization of isolates, symptoms, transmission and epidemiology, chemical and biological control of the virus reviewed by Karthikeyan et al. [5]. The use of mungbean cultivars resistant to MYMV has long been considered an effective and economical way to control the virus [6]. Many studies evaluated the mungbean germplasm for resistance to MYMV. However, only a few germplasm were found to be resistant [4,7,8,9]. Despite the advances in deciphering the mungbean genome, limited information is known about the genes and mechanisms underlying mungbean resistance to MYMV, which is essential for developing effective control methods. Therefore, comprehensive knowledge of mungbean responses to MYMV infection is needed for developing methods for the management of virus. 

Mungbean responses to the virus are complex and associated with the numerous biological and physiological processes involving the up- or down-regulation of genes. The discovery of the differentially expressed genes (DEGs) regulating the mungbean defense response to MYMV is vital in understanding genes and molecular mechanisms associated with resistance. This information is helpful for mungbean researchers to understand the complex interactions between mungbean and the virus. Taking advantage of second and third-generation sequencing technologies, comparative transcriptome analysis through RNA sequencing (RNA seq) is the most popular method for detecting DEGs between two models [10,11,12,13]. Moreover, transcriptome analysis provides detailed information to understand the dynamics of interaction between the host and pathogen [14,15]. By comparing the transcriptome data from the pathogen-infected and control, countless studies have been attempted to elucidate the complete details of the genes and pathways involved in the molecular mechanism of resistance to pathogens [16,17,18].

Recently, transcriptome comparisons among resistant and susceptible genotypes to pathogens in various crops, including mungbean [19], urdbean [20,21], soybean [22,23], rice [24,25], and tomato [16] extended our knowledge on the genes and mechanisms underlying the resistance to plant pathogens. In regard to the available literature information, RNA-Seq has yet to be used to investigate the transcriptome response of mungbean to MYMV. In the present investigation, the transcriptome response of two mungbean genotypes to MYMV was analysed using RNA seq. Our study identified specific and mutual DEGs and unveiled the different responses to MYMV infection in these two mungbean genotypes. This information has given new insights into MYMV-resistant and paved the way for breeding MYMV resistance in the future.

## 2. Materials and Methods

### 2.1. Genotype Panel and Pathogen Inoculation

Two mungbean genotypes were received from Agricultural Research Station, Tamil Nadu Agricultural University, Virinjupuram, India, i.e., MYMV-resistant genotype ‘VGGRU-1’ and the susceptible genotype ‘VRM (Gg) 1” [4]. Healthy seeds of both genotypes were agroinoculated with infectious virus construct VA 239 (KA30 DNA A + KA27 DNA B) [26] and raised in a plant growth chamber at ideal condition [Temperature (25 °C), relative humidity level (60–70%) and light/dark photoperiod (16/8  h)]. Agroinoculation, assessment of MYMV symptoms, and virus detection was performed following the procedure detailed by Karthikeyan et al. [6] and Sudha et al. [4,27]. The agroinoculation screening of mungbean genotypes was completed thrice.

### 2.2. Library Preparation and Illumina Sequencing

Leaves were collected at mock and inoculated plants at 25- and 40-days post-inoculation (DPI) and finely ground using liquid nitrogen and stored at −70 °C. For the construction of the cDNA libraries, the 25 and 40 DPI leaves were pooled together for RNA isolation and made as an infection library following the pooled cDNA library construction procedure [28], while leaves were uninoculated (Control) served as a non-infection library. Total RNA was isolated by the RNeasy plant mini kit (Qiagen, Hilden, Germany) following the user guidelines and treated with RNase-free DNAseI (Promega, Madison, WI, USA). RNA quantity and quality were determined by the bio spectrometer (Eppendorf, Hamburg, Germany) based on the absorbance ratio at 260 nm and 280 nm. The RNA integrity value (RIN) of the samples was confirmed by the Agilent Bioanalyzer 2100 system (Agilent Technologies, Santa Clara, CA, USA). Four cDNA libraries derived from VGGRU-1 control, VGGRU-1 infected, VRM (Gg) 1 control, and VRM (Gg) 1 infected were constructed by Illumina TruSeq RNA sample preparation kit following the user guidelines (Illumina Inc., SanDiego, CA, USA). Four cDNA libraries were sequenced by IlluminaHiSeq2000 platform with paired-end (PE) reads of 101 bp at Phyzen Genomics Institute, Seoul, South Korea.

### 2.3. Reads Filtering and Mapping 

Before alignment, raw reads obtained from each library were filtered to get clean high-quality reads by removing low-quality reads. The clean high-quality reads were obtained by following the method with three steps: in the first step, bacterial contaminants were removed from raw reads by mapping onto the available bacterial genomes through a burrows wheeler aligner (BWA) [29]. The second step included PCR copies, and ribosomal (rRNA) reads filtering by FastUniq [30] and SortMeRNA [31], respectively, and the uncompromising quality control and taking out the adapter contamination by NGS QC Toolkit (v2.3.3) was followed in the third step [32]. The clean high-quality reads were mapped to the mungbean reference genome (https://legumeinfo.org/genomes/gbrowse/Vr1.0; accessed on 6 February 2018) using burrows-wheeler aligner (BWA) [29] with default parameters.

### 2.4. Analysis of Differentially Expressed Genes

The expression levels of the gene transcripts were measured using fragments per Kilobase per million (FPKM) values estimated by RNA-Seq by Expectation-Maximization (RSEM) [33]. DEGs in VGGRU-1 control vs VGGRU-1 infected, and VRM (Gg) 1 control vs VRM (Gg) 1 infected were identified by Bioconductor package edgeR [34] using the threshold of false discovery rate (FDR) of ≤0.001 and the absolute value of Log2 fold-change ≥2. DEGs were signified in Venn diagrams using VENNY v.2.1 (http://bioinfogp.cnb.csic.es/tools/venny/; accessed on 18 August 2018). To investigate the function of the DEG transcripts, gene ontology (GO) analysis was conducted by the BLAST2GO software program (http://www.blast2go.org; accessed on 11 December 2018) with the default parameter. The main GO categories to which the DEGs be appropriate were confirmed next, and then genes were subject to BLAST, mapping, and annotation.

### 2.5. Quantitative Real-Time PCR (qRT-PCR) Analysis

Based on the functional annotation importance, seven genes related to plant defense response to pathogen infection were chosen for validation through quantitative real-time PCR (qRT-PCR). Primer pairs from the gene sequences were designed using Primer 5.0, and the primer specificity was verified by blasting the sequences at National Center for Biotechnology Information (NCBI) database. For normalizing the gene expression level, ubiquitin was used as a reference gene. Total RNA was isolated by RNeasy plant mini kit (Qiagen, Hilden, Germany). Consequently, the DNA-free RNA was used for first-strand cDNA synthesis by transcriptor First Strand cDNA Synthesis Kit (Roche Applied Science, Penzberg, Germany) following the manufacturer’s instructions. PCR amplification was performed using 2× SYBR Green PCR Master Mix (TaKaRa, Kusatsu, Shiga, Japan) and Light-Cycler^®^ 480 (Roche Applied Science, Penzberg, Germany) following the standard protocol. Three independent replicates were completed, and each gene’s relative expression was measured by the comparative 2^-ΔΔCt^ method.

## 3. Results

### 3.1. Mungbean Genotypes Reaction to MYMV

**Figure 1:**
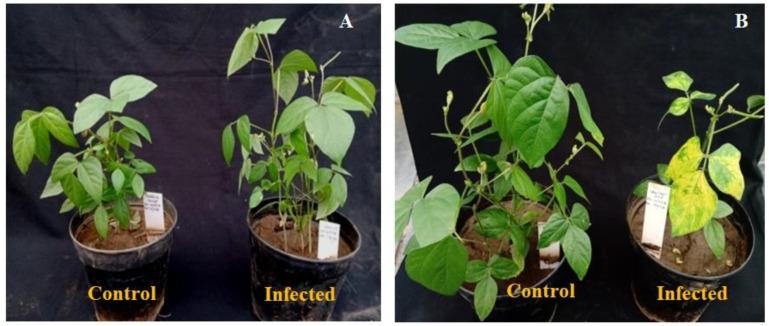
Development of symptoms of MYMV in the agroinoculated plants (**A**) VGGRU-1 and (**B**) VRM (Gg) 1.

Two mungbean genotypes, VRM (Gg) 1 and VGGRU-1, were inoculated with MYMV, and symptoms were observed in uninfected control and infected plants. A typical mosaic symptom was observed in susceptible mungbean genotype VRM (Gg) 1. On the contrary, there were no visible symptoms on the resistant genotype VGGRU-1 until 40 DPI (Figure 1). The viral DNA was detected in inoculated plants using PCR analysis of the coat protein gene of MYMV, while absent in uninfected control plants. The response of VRM (Gg) 1 and VGGRU-1 after infection with MYMV was as expected; thus, it is meaningful to use their leaves for further transcriptome analysis.

### 3.2. Summary of Transcriptome Data Set

We sequenced four cDNA libraries (VGGRU-1 control, VGGRU-1 infected, VRM (Gg) 1 control, and VRM (Gg) 1 infected) and generated a total of 95.8 million raw reads ranging from 23.2 to 25.1 million raw reads per library. All the raw reads were submitted to the NCBI database (Sequence Read Archive (SRA) with the accession number PRJNA742191). The read length from each library was 101 bp. GC percentage of the sequence data was about 43% in four libraries. Further, we used stringent criteria to filter the clean high-quality reads and aligned more than 80% reads based on the reference genome and used for DEG analysis. 

### 3.3. DEGs in Response to MYMV Infection

**Figure 2:**
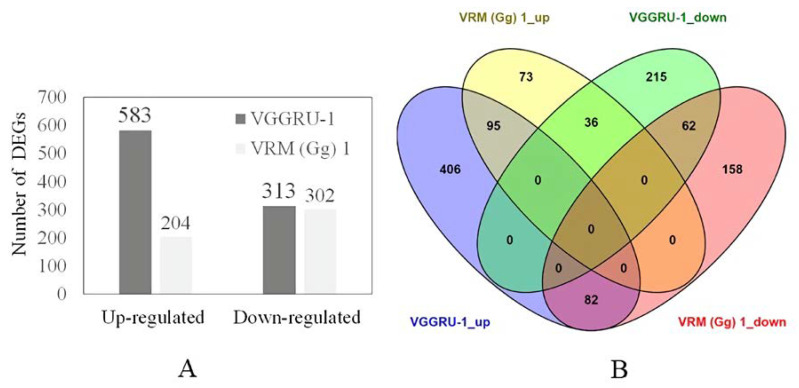
Differentially expressed genes (DEGs) identified through RNA-Seq analysis in resistant (VGGRU_1) and susceptible (VRM (Gg) 1) genotypes upon MYMV infection. (**A**) The total number of up- and down-regulated DEGs identified in resistant and susceptible genotypes. (**B**) A Venn diagram depicting the number of DEGs expressed in resistant and susceptible genotypes after MYMV infection.

We used the criteria of false discovery rate (≤0.001) and fold change greater than or equal to identify DEGs in resistant and susceptible genotypes. The total number of DEGs was greater in VGGRU-1 than in VRM (Gg) 1. The Venn diagram shows the distribution of DEGs in both genotypes (Figure 2). There were 896 DEGs between the VGGRU-1 control and infected samples. Among them, 583 genes were upregulated, and 313 genes were down-regulated (Tables S1 and S2). Likewise, 506 DEGs were detected between the VRM (Gg) 1 control and infected samples. Of these, expression levels of 204 genes were upregulated, and 302 genes were down-regulated (Tables S3 and S4). A total of 275 DEGs were common between VGGRU-1 and VRM (Gg) 1. The number of up and down-regulated genes in both genotypes was 95 and 62, respectively. Notably, in response to MYMV infection, 82 upregulated genes in VGGRU1 were downregulated in VRM (Gg) 1, and 36 downregulated genes in VGGRU-1 were upregulated in VRM (Gg) 1.

### 3.4. Gene Ontology Analysis of DEGs

**Figure 3:**
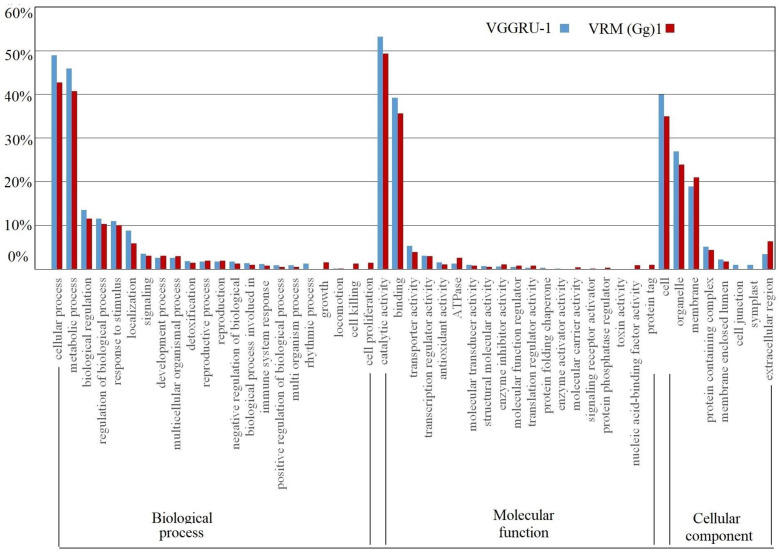
Functional categories of differentially expressed genes in the resistant (VGGRU-1) and susceptible (VRM (Gg) 1) genotypes. The percentage of DEGs is grouped into one of three main gene ontology (GO) categories viz., biological process, cellular component, and molecular function.

Differentially expressed genes identified after MYMV infection were classified into one of the three GO categories: biological processes (BP), molecular function (MF), and cellular components (CC). The DEGs with the unknown function were not related to any GO categories taken as novel genes responding to MYMV infection. In the biological processes category, most of the DEGs were related to the cellular process, metabolic process, biological regulation, regulation of the biological process, response to stimulus, localization, and signaling (Figure 3). The amount of DEGs related to the class of cellular and metabolic processes was higher in the resistant genotype (49 and 45%) compared to the susceptible genotype (42 and 41%), whereas DEGs in the biological regulation, regulation of the biological process, response to stimulus, localization, and signaling was marginally higher in the resistant genotype than that in the susceptible genotype. Besides, some of the DEGs were mainly related to cell killing and proliferation, and growth in the susceptible genotype, whereas several DEGs were related to the rhythmic process in the resistant genotype. On the other hand, the molecular function category large number of DEGs were associated with the catalytic activity, binding activity, transporter activity, transcription regulator activity, and antioxidant activity. Protein tag- and nucleic acid-binding factor activity-related DEGs were detected only in the susceptible genotype. Likewise, DEGs are related to protein folding chaperone, enzyme activator activity, and toxin activity specific to resistant genotype. Eventually, in the cellular components category, many DEGs were related to the proteins that are localized in the cell, organelle, membrane, protein-containing complex, membrane-enclosed lumen, and extracellular region. Some DEGs were solely related to cell junction and symplast in the susceptible genotype. These results collectively show the vital role of these GO categories in the MYMV resistance of the mungbean.

### 3.5. Analysing DEGs Related to Defense Response to Pathogen Infection

**Figure 4:**
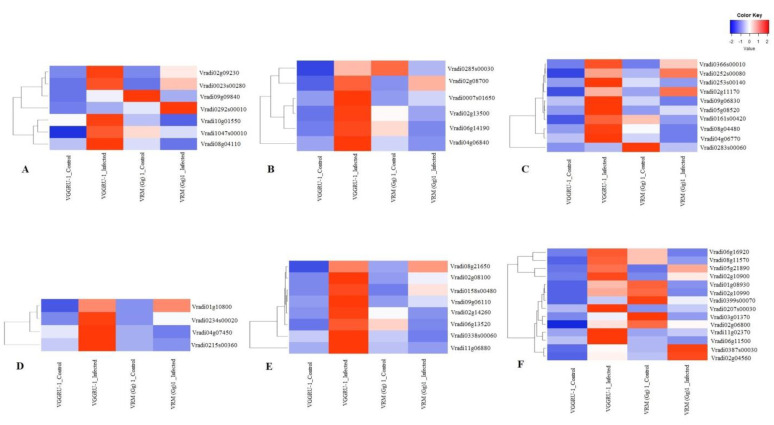
Heat map of several DEGs groups (**A**,**B**) defense and pathogenesis, (**C**) receptor-like kinases; serine/threonine protein kinases (STKs), (**D**) hormone signaling, (**E**) transcription factors, and chaperons, and (**F**) secondary metabolites.

Though many differences among the resistant and susceptible genotypes were unveiled by the DEGs and GO analysis, we were mainly focused on common DEGs (275) between the resistant and susceptible genotypes (Table S5). Of these, several DEGs were found to be related to the plant defense response to pathogen infection, and they belonged to the following categories; defense and pathogenesis, receptor-like kinases; serine/threonine protein kinases (STKs), hormone signaling, transcription factors, and chaperons, and secondary metabolites Figure 4 and Table S6. Here, we outlined a few examples for each category; defense and pathogenesis-related DEGs; TIR/CC-NBS-LRR disease resistance protein (*Vradi02g09230* and *Vradi10g01550*), and LRR and NB-ARC domain disease resistance protein (*Vradi08g04110*), disease resistance-responsive (dirigent-like protein) family protein (*Vradi08g01660*), protein phosphatase 2C family protein (*Vradi06g14190*), small GTP-binding protein (*Vradi04g06840*), and DEAD-box ATP-dependent RNA helicase-like protein (*Vradi02g13500*). Receptor-like kinases (RLK); serine/threonine protein kinases (STKs) related to DEGs (*Vradi04g06770, Vradi08g04480,* and *Vradi09g06830*). Hormone signaling-related DEGs, including jasmonic acid carboxyl methyltransferase (*Vradi04g07450*) and ethylene-responsive transcription factor (*Vradi0215s00360*). Transcription factors and chaperons related to DEGs including, WRKY (*Vradi06g13520*), bHLH (*Vradi02g14260*), MYB (*Vradi08g21650*), and HSP (*Vradi11g06880*). Secondary metabolite-related DEGs such as terpene synthase (*Vradi02g10990*), galactinol synthase (*Vradi08g11570*), β-galactosidase (*Vradi06g16920*), UDP-Glycosyltransferase superfamily protein (*Vradi02g04560*), Cytochrome P450 superfamily protein (*Vradi01g08930*), and alcohol dehydrogenase (*Vradi06g11500*)**.**

### 3.6. Validation of Defense Response-Related DEGs

**Figure 5:**
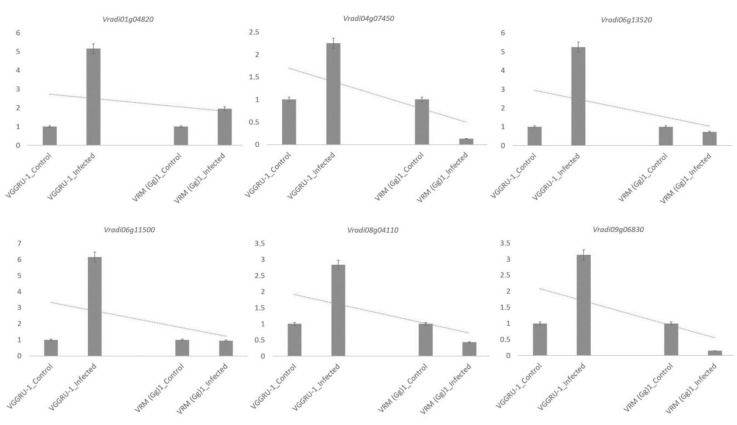
Graphs denote qRT-PCR-based validation of DEGs of mungbean leaves infected with mungbean yellow mosaic virus. The y-axis denotes relative fold change compared to control and infected. Data represent means ± SD of three replicates.

At least one representative DEG from each functional category was selected for qRT-PCR analysis to confirm the expression pattern of DEGs identified by RNA-Seq. A total of six DEGs, including *Vradi08g04110*, *Vradi09g06830*, *Vradi04g07450*, *Vradi06g13520, Vradi06g11500*, and *Vradi01g04820,* were used for qRT-PCR analysis. The results were consistent with the RNA-Seq results and had almost similar expression patterns in all the analysed DEGs. Overall, these data gave a detailed picture of how these DEG’s expression patterns occurred in the resistant and susceptible genotypes. Figure 5 shows the expression of the DEGs in the resistant and susceptible genotypes after MYMV infection. 

## 4. Discussion

The publically accessible mungbean whole genome sequences, integrated with the second-generation RNA seq platform, offered a potential method for investigating the DEGs in mungbean in response to pathogen infection. YMD in the mungbean and its relatives, including blackgram and cowpea, is mainly caused by MYMV and MYMIV. Transcriptomics analysis of resistant and susceptible genotypes in response to MYMIV was investigated in blackgram and mungbean [19,21]. It revealed the major genes and metabolism pathways linked to the disease resistance. However, no such investigation on the interaction of the mungbean and MYMV has been published. Previously, in our lab, we identified that mungbean genotype VGGRU-1 is resistant to MYMV, while VRM (Gg) 1 is susceptible [4]. In the present investigation, we prolonged the fundamental understanding of mungbean response to MYMV infection by comparing the transcriptome changes between VGGRU-1 and VRM (Gg) 1. Since MYMV is not transmitted mechanically, whiteflies-based inoculation cannot assure a 100% infection rate. Therefore, we used an infectious clone of MYMV (VA 239) to infect the mungbean genotypes using the agroinoculation method. This method provides the systemic infection compared to previous studies [19,21], which used the viruliferous whiteflies to inoculate the resistant and susceptible genotypes, and analysed the transcriptome changes. We used the RNA seq approach to obtain the transcriptomes of MYMV infected and uninfected control of VGGRU-1 and VRM (Gg) 1. The number sequence reads from four libraries ranged from 23.2 to 25.1 million reads, and more than 80% clean high quality reads matched on the reference genome, which was similar to reported mungbean RNA-seq data [35,36,37], confirming that the sequencing depth was satisfactory for the transcriptome coverage and further data analysis. We have found 896 genes in resistant genotype and 506 genes in susceptible genotype differentially expressed between their respective infected and uninoculated control. The number of DEGs in the resistant genotype VGGRU-1 was higher than the susceptible genotype, VRM (Gg) 1, following virus infection. This finding is consistent with the reports of Kundu et al. [21] and Dasgupta et al. [19], who detailed that resistant lines had greater DEGs than the susceptible. GO analysis found that pathogen response was related to the DEGs involved in transcription factor activity, hormone signaling, protein kinase activity, and metabolic process. Despite VGGRU-1 and VRM (Gg) 1 having different MYMV infection responses, 275 common DEGs were found in both mungbean genotypes. Previous studies have shown the advantage of focusing on the common DEGs between the resistant and susceptible genotypes to discover the possible candidate resistance genes in various diseases [15,22]. Therefore, we were mainly focused on 275 common DEGs. Among them, several DEGs were found to be related to the plant defense response to pathogen infection, and they belonged to the following categories; defense and pathogenesis, receptor-like kinases; serine/threonine protein kinases (STKs), hormone signaling, transcription factors, and chaperons, and secondary metabolites.

### 4.1. Defense and Pathogenesis Related Genes

Plant disease resistance genes (R genes) are important components of the genetic resistance mechanism in plants. In the recent past, many plant R genes exhibiting resistance to viral pathogens have been identified and cloned in various crops. The majority of R genes in plants encode CC/TIR-NBS-LRR and LRR and NB-ARC disease resistant proteins. Two DEGs *Vradi09g09840* and *Vradi1047s00010* encoding domain of CC-NBS-LRR, *Vradi10g01550* encoding domain of TIR-NBS-LRR, and *Vradi08g04110* encoding domain of LRR and NB-ARC disease resistant proteins differentially expressed. These genes were markedly reversed in response to resistant (Up-regulated) and susceptible (Down-regulated) genotypes following virus infection. Besides, three DEGs (*Vradi0023s00280*, *Vradi02g09230,* and *Vradi0292s00010*) encode TIR-NBS-LRR and CC-NBS-LRR were higher up-regulation in resistant mungbean genotype compared to the susceptible genotype upon infection. This was in agreement with Li et al. [38], and Karthikeyan et al. [39], who reported that the up-regulation of CC/TIR-NBS-LRR and LRR and NB-ARC genes following infection of soybean with soybean mosaic virus was related to resistance.

Disease resistance-responsive (dirigent-like protein), small GTP-binding proteins, and protein phosphatase 2C family proteins are related to disease resistance in plants. DIR proteins and disease resistance response family proteins have a similar dirigent-conserved domain. They play a major role in arbitrating the free radical coupling of monolignol plant phenols in plants to yield lignans and lignins; therefore, DIRs have been involved in disease resistance responses. *Vradi08g01660* encoding disease resistance-responsive (dirigent-like protein) family protein up-regulated in resistant genotype. Similarly, the involvement of dirigent genes and their apparent upregulated expression in response to attacks by pathogens [40,41,42] is of particular interest. The small GTP-binding gene families are related to the signal transduction in plants. It involves GTPase activity and activates the protein kinases and LRR related to disease resistance [43]. The up-regulation of DEG *Vradi04g06840* encoding Small GTP-binding protein in the resistant genotype compared to the susceptible genotype underlines the importance of this gene during plant disease resistance. 

Protein phosphatase 2C family proteins have been important in regulating the abscisic acid signaling pathway and adaptation to environmental stresses. In our study, upregulation of two DEGs (*Vradi02g08700* and *Vradi06g14190*) encoded protein phosphatase 2C was observed in resistant genotype; in contrast, lower-level expression compared to resistant genotype or downregulation was seen in susceptible genotype. Protein phosphatase 2C involves disease resistance by activating defense response in tobacco and soybean [44,45]. The upregulation of protein phosphatase 2C showed ABA-induced functions as a key regulator of *Rsv3*-mediated soybean mosaic virus resistance, limiting virus spread in soybean [45]. The involvement and significance of DEAD-box RNA helicases, which is the major family of RNA helicases, are known for their role against pathogens in plants [46]. Therefore, the expression levels of three DEGs belonging to the DEAD-box RNA helicases family were examined in the present study. All three DEGs were upregulated in resistant genotype, suggesting their involvement resistance. Notably, *Vradi0285s00030* and *Vradi02g13500* were upregulated in the resistant genotype, while they were down-regulated in the susceptible genotypes. *Vradi0007s01650* expression level was highly up-regulated in the resistant genotype, whereas only a slight up-regulation was observed in the susceptible genotype after virus infection. Li et al. [47] showed that overexpression of *OsBIRH1* encode DEAD-box RNA helicases in transgenic Arabidopsis plants resulted in an increased expression of defense-associated genes and improved the disease resistance as well as oxidative stress tolerance. 

### 4.2. LRR-RLK/STK Genes

LRR-RLKs are important components in regulating hormone signaling, abiotic and biotic stress responses in plants. STKs are receptor proteins that facilitate the signal transduction in plant defense responses [48,49]. It is mainly involved in identifying and transduction of pathogen-derived signals at the time of plant and microbe interactions. Several DEGs (*Vradi0161s00420, Vradi0253s00140, Vradi0283s00060, Vradi04g06770*, *Vradi08g04480*, and *Vradi09g06830*) from this family is upregulated in resistant genotype while downregulated in susceptible genotype. Although, several DEGs (*Vradi0252s00080*, *Vradi02g11170, Vradi0366s00010*, and *Vradi05g08520*) are highly up-regulated in the resistant genotype, but only a slight up-regulation was detected in the susceptible genotype after virus infection. Previous studies showed that overexpression of this gene family is related to disease resistance and defense responses, emphasizing important amino acids in specific regions, which are closely associated with plant disease resistance signal transmission [50,51].

### 4.3. Genes Involved in SA, JA, and ET Pathway

Plants can develop refined defense systems to prevent themselves against pathogen infection and cope with pathogen invasion by triggering numerous defense pathways. Pathogen infection induces the level of hormones such as salicylic acid (SA), jasmonate (JA), and ethylene (ET). These hormones are determined by the pathogen and play a major role in developing a strong defense system [52,53]. The plant hormone JA and its derivatives have been identified as important regulators in a plant’s immune system, playing critical roles in pathogen defense responses [54]. *Vradi04g07450* encoded S-adenosyl-L-methionine: jasmonic acid carboxyl methyltransferase (JMT). JMT involves the conversion of JA to MeJA, and overexpression of the JMT gene in transgenic Arabidopsis and rice plants induces the constitutive expression of JA-responsive genes and regulates the plants to defend themselves against infection by pathogens and herbivores [55,56]. Besides, one DEG *Vradi0234s00020* encodes allene oxide synthase (AOS) upregulated in both genotypes during virus infection, but up-regulation in resistant genotype was slightly high compared to susceptible genotype. AOS is the first enzyme in the branch pathway leading to the biosynthesis of JA, and studies reported that expression of AOS determines defense gene activation in tomato [57]. Two DEGs (*Vradi0215s00360* and *Vradi01g10800*) belong to the ethylene-responsive transcription factor (ERF) family regulated during virus infection. ET has been shown to regulate the expression level of pathogenesis-related genes via ERFs in earlier studies, ERFs were likely to have a role in the control of plant defense mechanisms by acting as transcriptional activators or repressors of GCC-box mediated gene expression [58,59,60,61]. 

### 4.4. Transcription Factors and Secondary Metabolites 

Transcription factors WRKY, MYB, and bHLH families have been known for their role in an elaborate regulation network by interacting with target genes in plants. Additionally, they are related to activation of defense gene expression and regulation of phytohormones crosstalk [62,63,64,65,66,67]. In this study, three types of transcription factors, including three WRKYs, two bHLH, and two MYB, were differentially expressed. WRKY proteins are an important family of transcriptional regulators identified solely in plants. They have been implicated in the plant’s response to biotic stress in different ways, including as transcriptional activators or repressors [68]. We have identified three DEGs belonging to WRKYs, i.e., *Vradi06g13520*, *Vradi0338s00060*, and *Vradi0158s00480,* differentially expressed following virus inoculation. In particular, *Vradi06g13520* and *Vradi0338s00060* showed a 2–5-fold increase in resistant genotype and a 2–3-fold decrease in susceptible genotype. *Vradi0158s00480* is upregulated in resistant genotype higher than susceptible genotype. So far, many researchers have discussed the involvement of WRKYs in plant defense response against various pathogens, including MYMIV, and how WRKYs interact with their target genes or crosstalk with genes involved in plant hormone signaling such as SA, JA, and others [69]. The bHLH proteins belong to a class of superfamily transcription factors, which can bind to particular target sites in DNA. Increasing evidence indicates that bHLHs regulate plant defense responses to pathogens [66,70]. Two DEGs (*Vradi02g14260* and *Vradi09g06110*) encoded bHLH showed up-regulation following virus infection in resistant genotype. In the susceptible genotype, *Vradi02g14260* showed downregulation while *Vradi09g06110* expressed a comparatively low level than resistant genotype. MYB proteins are reportedly involved in several functions, including pathogen resistance [67]. Two DEG (*Vradi02g08100* and *Vradi08g21650*) showed upregulation in both genotypes following virus infection. Ibraheem et al. [71] reported that expression of MYB induces 3-deoxyanthocyanidins and enhances resistance against leaf blights in maize. The small heat shock proteins (sHSPs) and the related a-crystallins are virtually ubiquitous proteins that are strongly induced by various other stresses in prokaryotic and eukaryotic cells. sHSPs were shown to be involved in the defense response against *Ralstoniasolanacearum* in *Nicotiana* plants [72]. In this study, *Vradi11g06880* encoded HSP21 showed differentially expressed. HSP21, a nuclear-encoded chloroplast-localized sHSP, has been described for its role in stress tolerance in plants [73]. 

Many secondary metabolites found in plants have a role in defense against herbivores, pests, and pathogens. In this study, we found that DEGs encoding terpene synthase (*Vradi02g10900*, *Vradi02g10990,* and *Vradi0399s00070*) involved in terpene metabolism, galactinol synthase (*Vradi08g11570*) involved in raffinose metabolism, β-galactosidase (*Vradi06g16920*), and UDP-glycosyltransferase superfamily protein (*Vradi0207s00030*, *Vradi0387s00030*, and *Vradi02g04560*) involved in glycosylation, cytochrome P450 superfamily protein (*Vradi01g08930*, *Vradi02g06800*, *Vradi03g01370,* and *Vradi11g02370*), β-glucosidase (*Vradi05g21890*), and alcohol dehydrogenase (*Vradi06g11500*) were reported to involved in plant defense mechanism against pathogens and insect pests infection [74,75,76,77,78,79,80,81,82]. Besides, we also found several DEGs belong to the family of formate dehydrogenase [83], chlorophyllase 1 [84], F-box family protein [85], methionine sulfoxide reductase [86], glutathione S-transferase family protein [87], and peroxidase superfamily [88] that are known to be involved indirectly or indirectly in plant defense mechanism against stress. 

### 4.5. Comparison of the Major DEGs with the Previous Reports of YMD Resistance in Mungbean

The information related to genes and molecular mechanisms of YMD resistance in mungbean is very limited. Two studies, Mathivathana et al. [89] and Dasgupta et al. [19], discussed the possible candidate genes associated with YMD resistance in mungbean. The former used the QTL mapping approach, and the latter used the RNA seq approach like our study. This study identified several DEGs linked to virus resistance, and they belonged to the gene families that have been reported by Mathivathana et al. [89] and Dasgupta et al. [19]. For instance, gene families including WRKY, bHLH, MYB S-adenosyl-L-methionine: jasmonic acid carboxyl methyltransferase, DEAD-box RNA helicases, small GTP-binding, cytochrome P450, and protein kinase superfamily protein/serine-threonine kinase. Mathivathana et al. [89] reported the major QTL (qMYMV4_1) governing YMD resistance at nucleotide positions Vr04:14504302 and15788321 on chromosome 4. Three genes *Vradi04g06770* (Protein kinase superfamily protein/serine-threonine kinase)*, Vradi04g06840* (small GTP-binding), and *Vradi04g07450* (S-adenosyl-L-methionine: jasmonic acid carboxyl methyltransferase) differentially expressed in our study were existed in this genomic region [89]. These genes are potential candidates to investigate MYMV resistance in the future. Although we have identified several DEGs from the gene families that are not previously reported in YMD resistance studies [19,21,89], for instance, DEGs belong to LRR and NB-ARC, and NBS-LRR gene families (*Vradi02g09230, Vradi08g04110, Vradi09g09840,* and *Vradi10g01550*), which are the largest group of plant R genes that play important roles in plant defense responses to various pathogens. Plant defense response is a complex system that requires comprehensive investigation stage by stage. This study only predicted the likely genes involved in MYMV resistance, and we do not have the functional validation of the identified genes. Therefore, before utilizing these genes for breeding purposes and understanding the specific roles of genes involved in the virus resistance, the identified genes from this study could be verified using the overexpression, gene knockout, or CRISPR approaches.

In summary, our study postulates the putative genes linked with resistance to MYMV. It revealed how the mungbean genes interact and respond to MYMV infection and cause resistance and susceptibility. We used RNA sequencing technology to compare the transcripts changes of control and infected resistant and susceptible genotypes in response to MYMV. Overall, the information generated from current research will be a valuable foundation for deciphering the molecular mechanism governing MYMV resistance in mungbean that can provide insights for future genetic breeding and facilitate the development of mungbean cultivars resistant against MYMV. 

## Data Availability

All fastq files have been submitted to the NCBI Sequence Read Archive database at https://www.ncbi.nlm.nih.gov/sra; accessed on 29 June 2021. NCBI accession for this project is PRJNA742191, and the SRA accession numbers: SRX11379693-SRX11379696.

## Supplementary Materials

The following supporting information can be downloaded at: https://www.mdpi.com/article/10.3390/pathogens11020190/s1, Table S1: List of DEGs up regulated in resistant genotype VGGRU-1; Table S2: List of DEGs down regulated in resistant genotype VGGRU-1; Table S3: List of DEGs up regulated in susceptible genotype VRM (Gg) 1; Table S4: List of DEGs downregulated in susceptible genotype VRM (Gg) 1; Table S5: List of common DEGs between resistant and susceptible genotypes and their annotation details; Table S6: Few examples for DEGs from different categories related to defense response.
